# Human gut microbiota: onset and shaping through life stages and perturbations

**DOI:** 10.3389/fcimb.2012.00144

**Published:** 2012-11-23

**Authors:** Lorenza Putignani

**Affiliations:** Parasitology Unit, Bambino Gesù Children's Hospital, IRCCSRome, Italy

Microbial taxa distribution and density of gut microbiota habitants diverge in different individuals and is modulated by diverse determinants of temporal and spatial variability. Until the moment of birth, the gastrointestinal (GI) tract of a normal fetus is almost sterile. During birth and thereafter, bacteria from mother and surrounding environment colonize the infant's gut by vertical and horizontal transmission. Rapidly after the birth, bacteria start to appear in the feces in a few hours and reach 10^8^–10^10^ per gram of faeces within a few days. The epithelium at the interface between intestinal microbiota and lymphoid tissue plays a critical role in shaping the mucosal immune response. When commensal/pathogenic bacteria homeostasis is broken up, the perturbation leads to immunological impairment and disease, crucial in “programming early phases” and dysbiosis establishment. Gut imbalance occurring during perinatal and neonatal life can lead to diseases such as irritable bowel syndrome (IBS), early-inflammatory bowel disease (IBD), respiratory and chronic pulmonary disease, immunological impairment, obesity and metabolic syndrome, hence triggering cardiovascular risks and nutritional impairment further along life stages. Investigation on individuality of gut microbiota onset and modulation requires to speculate on genetic and epigenetic affecting factors. Indeed, relationship between breast feeding, gut microbial taxa, and immune system response appears crucial in early life. However, classical microbiology is underpowered by its inability to provide unbiased representation of gut microbiota. Failure to cultivate *in vitro* the majority of microbiota taxa hampers a fulfilling description. The advent of high-throughput-omics-based methods, through the holistic view of the “systems biology,” is opening new avenues to the knowledge of the gut ecosystem. In the coming years, the plasticity of the gut microbiota will be even exploited to provide new categories of therapeutics, providing therapeutic modification of the gut microbiota, on the basis of specific microbe-microbe modulation and microbe-host interaction, aiming to correct and improve life style conditions, and medical management of chronic patients such as cystic fibrosis-affected people. Modern microbiology may really concur nowadays in addressing one of the most complicated challenges of the current medicine, the achievement of effective patient-tailored therapies to exactly depict the biology and physiology of the microbiota “organ” of each person. This volume aims to highlight several “frontiers” aspects of gut microbiota studies, through the contribution of 12 articles describing the microbiologist's, the “omics” people point of view, but also the clinician's complementary approaches coming from different expertises and research areas, such as obstetrics, neonatology and hepato-metabolic diseases. The first article, by Pessione (2012), presents a spectacular overview on Lactic Acid Bacteria (LAB), ancient microorganisms that, modulating sugar fermentation and decarboxylation/deimination, ensures their survival and colonization in the buffered environments of the GI trait, by a complex molecular cross-talk between LAB and host. LAB proteins, produced in response to gut, promote bacterial adhesion to mucosa and stimulate immune cells. Furthermore, LAB antagonistic relationships with other microorganisms constitute the basis for their anti-infective role. Thus, interesting perspectives for their utilization as antioxidant nutraceutical vectors are hypothesized. The second article, by Rigon et al. (2012) focuses on vertical determinants of gut variability associated to vaginal or cesarean delivery in the mother-child pair, and discuss breast- or formula feeding, also thoroughly discussed in the article by Guaraldi and Salvatori (2012). The two articles are particularly remarkable because they focus, by employing the point of view of the clinician, on the very early phases of gut microbiota programming, still “mysterious” and difficult to be unveiled. A cluster of three outstanding articles addresses, in a fascinating way, the gut “programming” and modification through life stages up to senescence (Kolling et al., 2012; Lagier et al., 2012; Ottman et al., 2012), with special emphasis on gut pathogens occurring during different ages (Kolling et al., 2012), or on data obtained by metagenome, metatranscriptome, and metaproteome integrated approaches (Ottman et al., 2012). Interestingly, Lagier et al. (2012), introduce the novel concept of “culturomics,” a breakthrough in gut microbiota research, with the microbial identification and characterization performed by MALDI-TOF technology. In the review by Kolling et al. (2012), the process of aging is discussed in term of changes due to environmental exposures that subsequently affect the immune system and host-associated microbiota. Within the host's shifting setting, infections by enteric pathogens likely exploit these shifts with resultant initiation of pathogenesis and/or establishment of a mutualistic relationship with the host leading to potential dissemination of the pathogen. There is still much to be learned about how life stages and perturbations shape the gut microbial population, and how these changes influence health and disease and predominant enteric pathogens. Future insights into the interdependency between environment-host-microbe network will be essential for development of novel therapeutic approaches that treat or prevent enteric disease. Understanding the roles of these factors within the host is complementary to external approaches (*e.g*., sanitation, water treatment) for controlling or eradicating pathogen dissemination. The paper by Berrilli et al. (2012) strengthen the approach undertaken by Kolling et al. (2012), on gut ecosystems and pathogenicity and deal with the inter-play between parasites and microbial gut ecology. The paper has been strongly desired in the e-book *Human gut microbiota: onset and shaping through life stages and perturbations*, because of the enormous novelty of the topic, still not properly approached by the current literature. Indeed, complex communities, different from bacterial gut ecosystems, including parasites (“parasitome”) and the even less studied fungi (“micetome”), surely co-evolve and interact with the gut microbiome modulating its ecology and physiology, reacting in a different way with and against the host. Indeed, interesting are the parasite immune-modulations at the gut enterocyte level on host immune reaction. On the other site, “omics” technology and their paramount effects on gut microbiota studies have been also deeply described in the articles by Shen et al. (2012) (genomics, metagenomics, and gut modifications), Vernocchi et al. (2012), and Masotti (2012) (gene expression, transcriptomics). Metabolic profiling has a wide potential to understand the complex interactions amongst components of the gut microbiota and to elucidate the relationships (cause/effect) between specific nutritional choices and related shifts in microbiota taxa composition. Particularly, the identification of gut metabolic signatures is extremely challenging, due to its linkage with nutritional choices. In fact, the whole set of metabolites, which can be detected in body fluids and characterizes the metabolic gut-associated phenotypes, can be named “metabotype” (Vernocchi et al., 2012). Dealing with a differential expression of miRNAs in different areas of GI tract can be considered as a function of microbiota composition. In these cases, intestinal microbiota are the “actors.” Conversely, we should also think to miRNAs as “actors” when, under proper conditions, influence the regulation of goblet-cell differentiation. Therefore, an interconnected cycle could be envisaged, as suggested by the Opinion article by Masotti (2012), where miRNAs and gut microbiota are the two main partners.

Finally, two remarkable papers by Alisi et al. (2012), and Manco (2012) open new avenues on non-alcoholic fatty liver disease (NAFLD), one of the most common causes of chronic liver disease worldwide, and on the gut-brain axis in obesity endophenotypes, respectively. In NAFLD, several observations suggest a potential role of microbiota in NAFLD development, authors suggest that probiotics, for their excellent tolerability, may affect gut microbiota, hence representing promising therapeutic agents to revert NASH-related liver damage. Furthermore, the interesting relationships between gut microbiota and developmental programming of the brain, discussed in the Opinion article by Manco (2012), are providing unpredictable evidence in studies on mental retardation, autism and role of gut dysbiosis through the route to the final outcome of the overt disease.

In summary, the articles herein presented, discuss data and matters concerning current investigation and future perspective on gut microbiota studies. In our opinion, further insights will come from interdisciplinary approaches progressively provided by enlarged consortia, including researchers and clinicians, able to exploit high-throughput technological platforms to apply translational workflows in diagnostic pipelines and, finally, in patient care and treatment programs.
